# Pannexin 1 binds β-catenin to modulate melanoma cell growth and metabolism

**DOI:** 10.1016/j.jbc.2021.100478

**Published:** 2021-02-26

**Authors:** Samar Sayedyahossein, Kenneth Huang, Zhigang Li, Christopher Zhang, Alexandra M. Kozlov, Danielle Johnston, Daniel Nouri-Nejad, Lina Dagnino, Dean H. Betts, David B. Sacks, Silvia Penuela

**Affiliations:** 1 Department of Anatomy and Cell Biology, Schulich School of Medicine and Dentistry, University of Western Ontario, London, Ontario, Canada; 2 Department of Laboratory Medicine, National Institutes of Health, Bethesda, Maryland, USA; 3 Department of Biology, Faculty of Science, University of Western Ontario, London, Ontario, Canada; 4 Department of Physiology and Pharmacology, Schulich School of Medicine and Dentristry, University of Western Ontario, London, Ontario, Canada; 5 Division of Experimental Oncology, Department of Oncology, Schulich School of Medicine and Dentistry, University of Western Ontario, London, Ontario, Canada

**Keywords:** malignant melanoma, PANX1, Wnt, β-catenin, mitochondria, pannexin, ATCC, American-type culture collection, BCA, bicinchoninic acid, DMEM, Dulbecco's modified Eagle medium, EDTA, ethylenediaminetetraacetic acid, KO, knockout, LEF1, lymphoid enhancer-binding factor 1, MBP, maltose-binding protein, MITF, microphthalmia-associated transcription factor, PANX1, pannexin 1, PBS, phosphate-buffered saline, TCGA, The Cancer Genome Atlas

## Abstract

Melanoma is the most aggressive skin malignancy with increasing incidence worldwide. Pannexin1 (PANX1), a member of the pannexin family of channel-forming glycoproteins, regulates cellular processes in melanoma cells including proliferation, migration, and invasion/metastasis. However, the mechanisms responsible for coordinating and regulating PANX1 function remain unclear. Here, we demonstrated a direct interaction between the C-terminal region of PANX1 and the N-terminal portion of β-catenin, a key transcription factor in the Wnt pathway. At the protein level, β-catenin was significantly decreased when PANX1 was either knocked down or inhibited by two PANX1 blockers, Probenecid and Spironolactone. Immunofluorescence imaging showed a disrupted pattern of β-catenin localization at the cell membrane in PANX1-deficient cells, and transcription of several Wnt target genes, including MITF, was suppressed. In addition, a mitochondrial stress test revealed that the metabolism of PANX1-deficient cells was impaired, indicating a role for PANX1 in the regulation of the melanoma cell metabolic profile. Taken together, our data show that PANX1 directly interacts with β-catenin to modulate growth and metabolism in melanoma cells. These findings provide mechanistic insight into PANX1-mediated melanoma progression and may be applicable to other contexts where PANX1 and β-catenin interact as a potential new component of the Wnt signaling pathway.

Pannexin 1 is a member of a glycoprotein family (PANX 1, 2, and 3) that oligomerizes to establish large pore channels between the intracellular and extracellular space for cell communication (1, 2, 3). Among Pannexins, PANX1 has been the primary focus of research because of its widespread expression (4). PANX1 mediates the release of small signaling molecules, such as adenosine tri-phosphate (ATP) (5). Additionally, intracellular PANX1 has been reported to function as a calcium leak channel in the endoplasmic reticulum (6, 7). PANX1 plays an important role in normal physiological processes, including skin development and wound healing as well as in pathophysiological conditions and metabolic disorders, such as Alzheimer’s disease, diabetes, inflammation, and cancer (8, 9). Melanoma is the most aggressive form of skin cancer with increasing incidence worldwide (10). There is growing interest in investigating the role of PANX1 in the regulation of cellular processes, such as proliferation, migration, differentiation, and invasion during melanoma tumorigenesis (11, 12). Currently, our understanding of the mechanisms through which PANX1 regulates cellular processes and the metabolic profile of melanoma cells is very limited. However, our recent findings indicate that knocking down (KD) PANX1 with shRNA in aggressive BL6 mouse melanoma cells, as well as in human melanoma cell lines, reduces the abundance of β-catenin (11, 12), a key transcription factor in the Wnt signaling pathway implicated in melanoma tumorigenesis (13). Wnt/β-catenin signaling regulates proliferation, migration, and invasion of melanoma cells (14, 15, 16). Additionally, immune evasion is a hallmark of melanoma progression (17), and active β-catenin signaling within melanoma tumor cells suppresses the recruitment of immune cells and contributes to melanoma immune evasion (18). Of note, β-catenin modulates aerobic glycolysis and regulates cancer cell metabolism (19), which may be an additional role of Wnt/β-catenin pathway in melanoma tumorigenesis. We postulated that PANX1 regulates melanoma cell metabolic profile, proliferation, and migration in part through cross talk with the Wnt signaling pathway. Here we showed that, PANX1 binds directly *via* its C-terminal region to β-catenin. Blocking or reducing PANX1 in melanoma cells decreases the levels of β-catenin and suppresses β-catenin transcriptional activity. Moreover, depletion of PANX1 attenuated the mitochondrial respiratory activity of melanoma cells. Our findings underline the molecular mechanisms through which PANX1 regulates melanoma tumorigenesis and suggests that PANX1 may be a new interactor in the Wnt signaling pathway and can potentially be a target for the treatment of malignant melanoma.

## Results

### Pannexin 1 associates with β-catenin in melanoma cells

**Figure 1:**
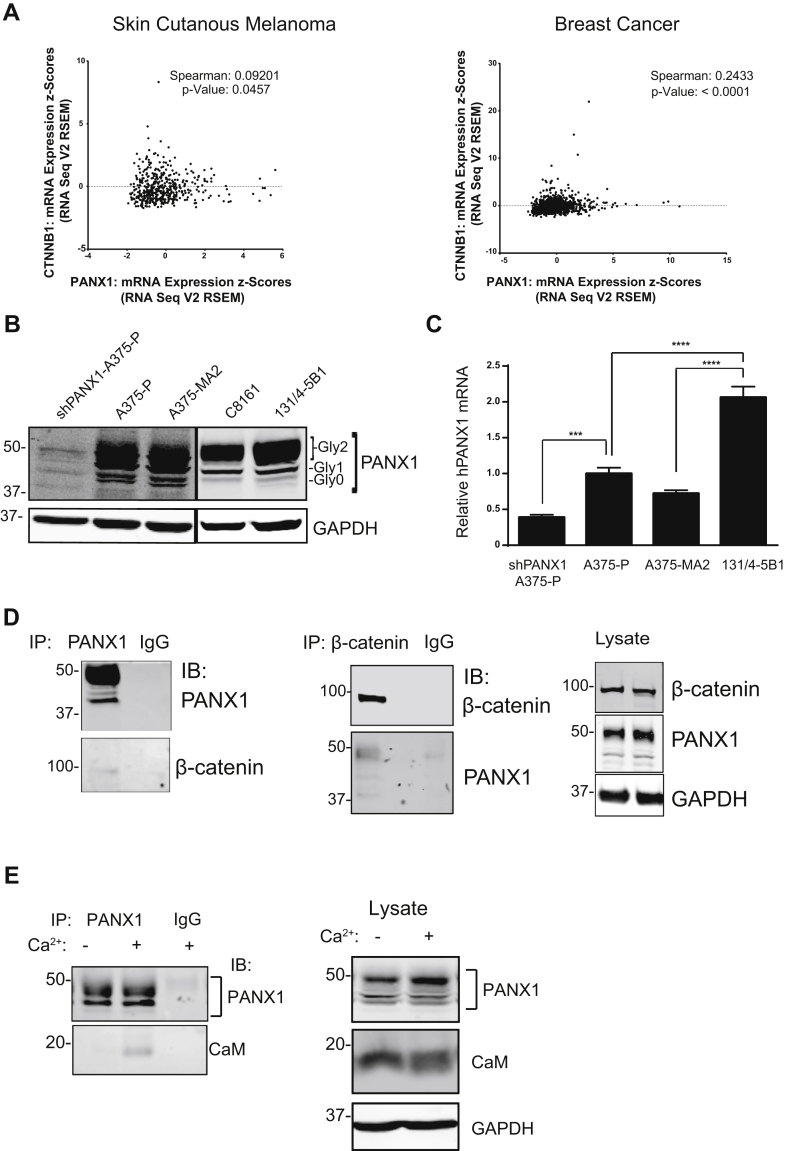
**PANX1 associates with β-catenin in melanoma cells**. *A*, analysis of PANX1 and β-catenin (CTNNB1) mRNA expression in melanoma and breast carcinoma in the Cancer Genome Atlas (TCGA) database revealed that there is a modest yet significant correlation between PANX1 and β-catenin mRNA expression levels in melanoma (*left panel*) and breast cancer (*right panel*). *B*, western blot of PANX1 protein expression among several malignant human melanoma cell lines showing abundant PANX1 expression in all cell lines, especially in 131/4-5B1 cells. Banding pattern of PANX1 shows different glycosylation states (Gly0, Gly1, and Gly2). A375-P cells transfected with shRNA against PANX1 (shPANX1-A375-P) were used as control to confirm antibody specificity. GAPDH was used as loading control. *C*, total RNA was extracted from the indicated cell lines. Human PANX1 mRNA was measured by quantitative RT-qPCR. The amount of mRNA was corrected to house-keeping gene (YWHAZ) as control in the same sample. Levels of mRNA in the A375-P cells were set to 1. The data represent the mean ± S.E. (*error bars*) of at least three independent experiments, each performed at least in three technical replicates (N = 3, n = 3). Statistical analysis conducted using one-way *ANOVA* with Tukey post hoc test ∗∗∗*p* < 0.001, ∗∗∗∗*p* < 0.0001. *D*, equal amounts of protein lysate (1 mg) from 131/4-5B1 cells were immunoprecipitated (IP) with anti-PANX1 (*left panel*) or anti-β-catenin antibodies (*middle panel*). Immuno-complexes were analyzed by SDS-PAGE. Lysates directly obtained from cells were loaded directly onto the gel (*Lysate*, *right panel*). Rabbit or mouse IgG antibodies were used as negative controls. Blots were probed with anti-PANX1 and anti-β-catenin antibodies. GAPDH was used as loading control. All data are representative of at least three independent experiments. Data show that PANX1 and β-catenin associate with each other in melanoma cells. *E*, equal amounts of protein lysate (1 mg) from A375-P cells were obtained with lysis buffer containing either 1 mM CaCl2 (+) or 1 mM EGTA (−). Lysates were immunoprecipitated (IP) with anti-PANX1 antibodies. Immune complexes were analyzed by SDS-PAGE. Blots were probed with anti-PANX1 and anti-calmodulin (CaM) antibodies (*left panel*). Mouse IgG antibodies were used as negative controls. Lysates directly obtained from cells were loaded onto the gel (*Lysate*, *right panel*). GAPDH was used as loading control.

To determine whether there is an interaction between PANX1 and β-catenin in melanoma cells, we first analyzed a panel of melanoma biopsies from the Cancer Genome Atlas (TCGA) and found a modest, yet significant, correlation between PANX1 and β-catenin mRNA (CTNNB1) in 471 patients with malignant melanoma (Fig. 1*A*, *left panel*). To investigate whether this correlation is restricted to melanoma or applies to other types of cancers, we analyzed TCGA data of 1108 biopsies taken from patients with breast cancer. We observed that, similar to melanoma, mRNA expression of PANX1 significantly correlates with that of β-catenin (Fig. 1*A*, *right panel*). Of note, both PANX1 and β-catenin have been implicated in survival of metastatic breast cancer cells and poor patient outcome (20, 21). Next, we evaluated the association of PANX1 and β-catenin in the melanoma cell environment. We assessed several human melanoma cell lines with transcriptional profiles similar to melanoma tumors (22) for their PANX1 expression (Fig. 1*B*). We observed that PANX1 is expressed in all cell lines tested but most abundant in 131/4-5B1 cells at both the protein and mRNA levels (Fig. 1, *B* and *C*). A375-P cells with PANX1 knockdown were used to validate the specificity of the antibody. We chose 131/4-5B1 cells to conduct immunoprecipitation studies, incubating protein lysates with specific antibodies against the carboxyl terminal region of PANX1. Endogenous PANX1 co-immunoprecipitated (co-IP) with β-catenin in 131/4-5B1 human melanoma cells (Fig. 1*D*, *left panel*). In contrast, no β-catenin precipitated with rabbit IgG, validating the specificity of the interaction (Fig. 1*D*, *left panel*). Reciprocal analysis revealed that endogenous PANX1 specifically co-IP with endogenous β-catenin from 131/4-5B1 human melanoma cells (Fig. 1*D*, *middle panel*).

PANX1 has been shown to act as a Ca^2+^-permeable channel at the endoplasmic reticulum (6, 7), providing a pathway for intracellular Ca^2+^ diffusion that controls several physiological processes from proliferation to apoptosis (5). The molecular nature and mechanism of this particular function of PANX1 are poorly understood. Many signaling pathways initiated by the rise in intracellular Ca^2+^ are mainly mediated by calmodulin, the master regulator of Ca^2+^ signaling in all eukaryotic cells (23). Calmodulin binds to several connexin forming gap junctions and modulates their functions in cell-to-cell transfer of metabolites (24, 25, 26). Given the similarity of connexin and pannexin structure, we investigated whether calmodulin interacts with PANX1 in melanoma cells. We immunoprecipitated PANX1 from A375-P and A375-MA2 cells in the presence of Ca^2+^ (Fig. 1*E*, Fig. S1*A*). Calmodulin co-IP with PANX1 in a Ca^2+^-dependent manner (Fig. 1*E*, *left panel*, Fig. S1*A*). The interaction between calmodulin and PANX1 in melanoma cells represents an additional layer of complexity in the modulation of Ca^2+^ signaling through PANX1.

**Figure 2:**
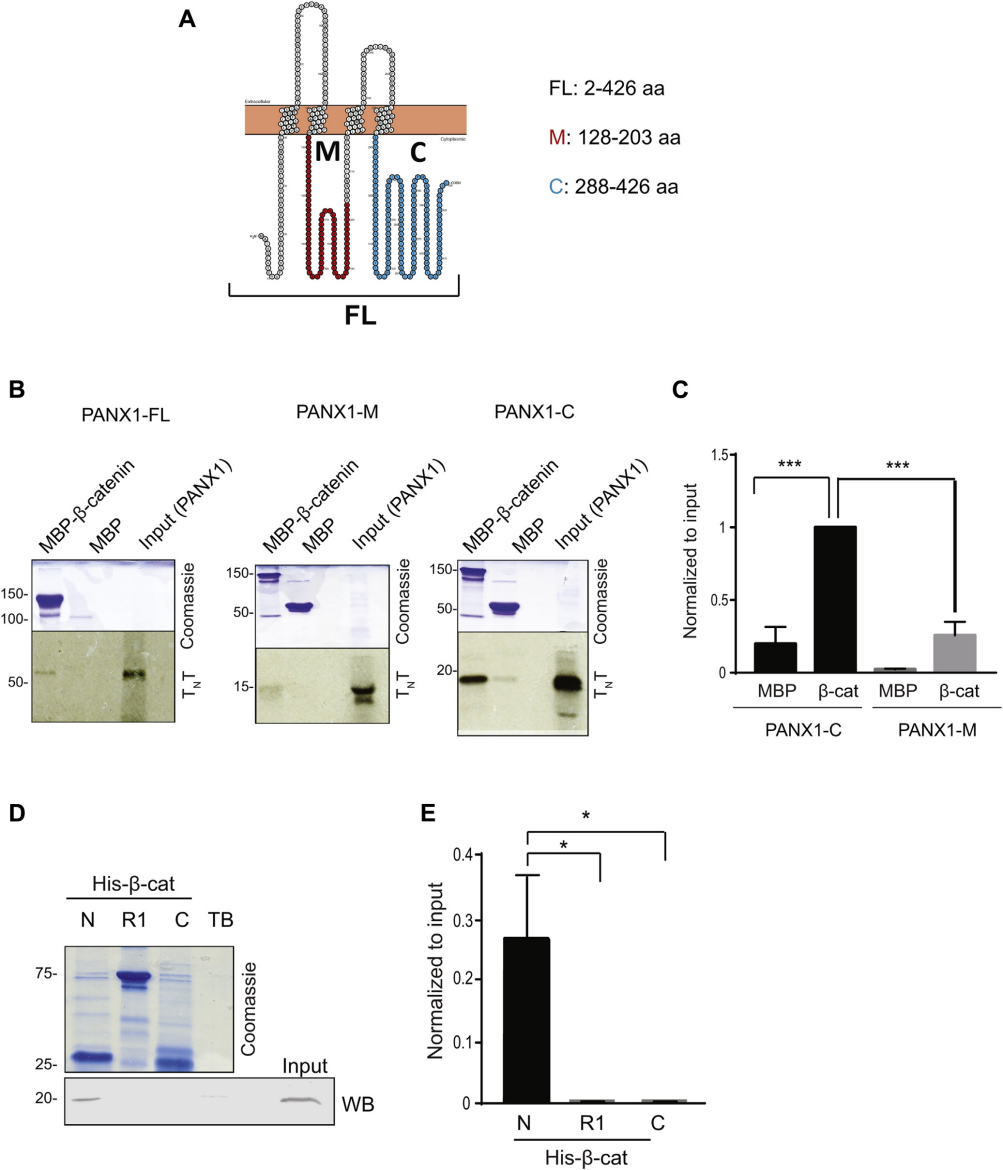
**C-terminal region of PANX1 binds directly to N-terminal region of β-catenin.***A*, schematic representation of PANX1 full-length (FL) and fragments M (*red*) and C (*blue*). The specific amino acid residues in each construct are indicated. *B*, [^35^S]methionine-labeled PANX1 fragments generated by T_N_T quick coupled transcription/translation system (Promega) and were incubated with equal amounts of maltose-binding protein (MBP)-β-catenin or MBP alone. Samples were resolved by SDS-PAGE. The gels were cut at the 75 kDa region and the *upper portion* was stained with Coomassie Blue (*upper panel*). The *bottom portion* of the gel was dried and analyzed by autoradiography (*lower panel*). “Input” depicts 5% of the T_N_T peptides used for the assay. The data are representative of at least three independent experiments. *C*, quantifications of blots in (*B*) using Image Studio V. 5.2. Data are normalized to the input and β-catenin sample in PANX1-C was set to 1. N = 3. Statistical analysis conducted using one-way *ANOVA* with Tukey post hoc test ∗∗∗*p* < 0.001. Data reveal that β-catenin directly binds to C-terminal region of PANX1. *D*, HEK293 cells were transfected with pcDNA3-myc-PANX1-C and lysed. Equal amounts of protein lysate were incubated with His-tagged portions of β-catenin (attached to Talon beads), namely N (AA 1–137), R1 (AA 138–781), or C (AA 666–781), or with Talon beads alone (TB, negative control). 1% of the lysate was loaded directly onto the gel (Input). Samples were resolved by SDS-PAGE and gels were cut at ∼25 kDa. The *lower portion* of the gels was transferred to PVDF and probed with anti-myc antibodies to detect PANX1-C (WB). The *upper portion* of the gel was stained with Coomassie blue. Data are representative of four independent experiments. *E*, quantifications of blots in (*D*) using Image Studio V. 5.2. Data are normalized to the input. N = 4. Statistical analysis conducted using one-way *ANOVA* with Tukey post hoc test ∗*p* < 0.05.

It is not possible to establish from the data shown in Figure 1*D*, whether PANX1 and β-catenin bind directly or through a protein complex. To address this question, we performed an *in vitro* analysis using a transcription and translation (T_N_T) system and purified recombinant proteins. Selected regions of PANX1 (Fig. 2*A*, *schematic*) were expressed using the T_N_T and labeled with [^35^S]methionine. The PANX1 constructs were incubated with Maltose-Binding Protein (MBP)-tagged β-catenin. After washing, complexes were resolved by SDS-PAGE, and gels were dried and processed by autoradiography. Analogous to findings with co-IPs (Fig. 1*D*), full-length PANX1 binds directly to purified β-catenin *in vitro* (Fig. 2*B*, *left blot*). Analysis of two fragments of PANX1 revealed minimal binding of the middle segment (M) of PANX1 (amino acids 128–203) to β-catenin (Fig. 2*B*, *middle blot* and Fig. 2*C*). In contrast, amino acids 288 to 426, which constitute the C-terminal segment (C) of PANX1, exhibit robust binding to β-catenin (Fig. 2*B*, *right blot*, and Fig. 2*C*). Binding specificity was confirmed by the absence of bands from samples incubated with MBP alone (Fig. 2, *B* and *C*). The expression levels of T_N_T products were comparable among samples (Fig. 2*B*). We investigated whether the C-terminal region of PANX1 (aa 288–426) is homologous to any other proteins. The results using NCBI blast analysis revealed that the 138 aa in the C-terminus region of PANX1, where β-catenin binds, is specific to PANX1 among different species (Fig. S2).

In addition, we conducted *in vitro* assays to identify the region of β-catenin where PANX1 binds. We conducted pull-down assays using selected β-catenin fragments, namely the N-terminal (amino acids 1–137), middle and C-terminal (amino acids 138–781, termed R1) and the C-terminal (amino acids 666–781) regions of β-catenin. His-tagged purified fragments of β-catenin were incubated with lysates of HEK293 cells expressing myc-PANX1-C. Complexes were isolated using Talon Metal Affinity Resin. PANX1 binds exclusively to the N-terminal region of β-catenin (amino acids 1–137) (Fig. 2, *D* and *E*). No binding is detected to the R1 or C constructs of β-catenin or to Talon beads alone. Collectively, these data demonstrate that the C-terminal region of PANX1 binds to the N-terminal portion of β-catenin.

### β-Catenin is reduced in PANX1-deficient melanoma cells

**Figure 3:**
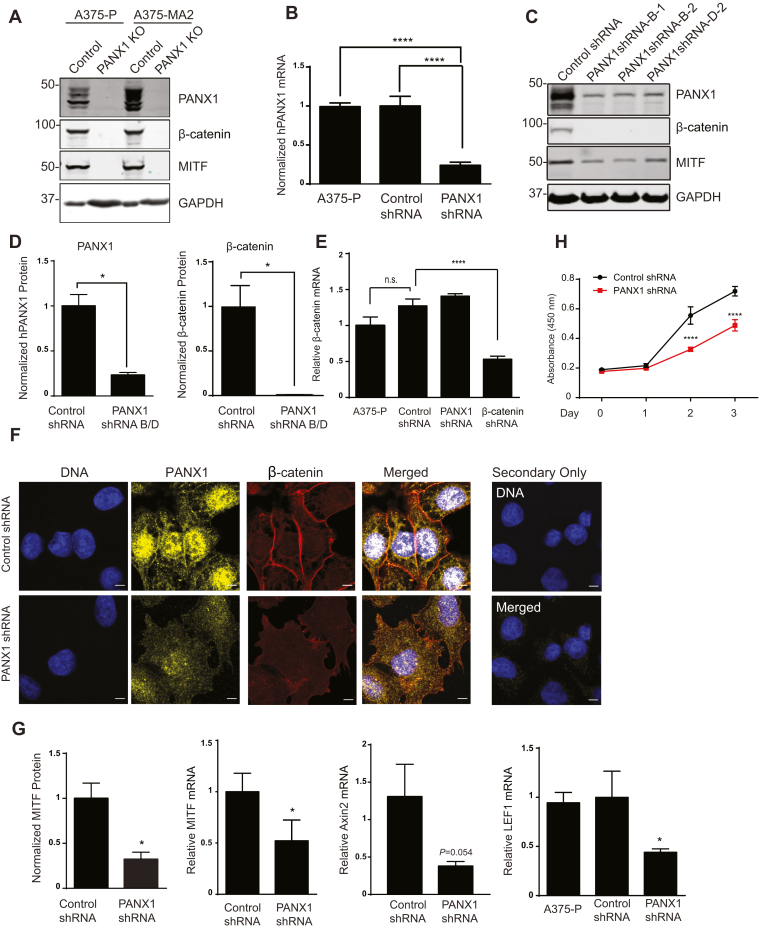
**β-catenin is depleted from PANX1-deficient melanoma cells.***A*, PANX1 was knocked out in A375-P and A375-MA2 cells by CRISPR/Cas9 system. Equal amounts of protein lysate from control and PANX1-depleted cells were resolved by SDS-PAGE. Western blotting was performed using the indicated antibodies. GAPDH was used as a loading control. *B*, total RNA was extracted from specified cells. Human PANX1 mRNA was measured by quantitative RT-qPCR. The amount of mRNA was corrected to YWHAZ in the same sample. mRNA in the A375-P cells was set to 1. The data represent the mean ± S.E. (*error bars*) of at least three independent experiments, each performed at least in three technical replicates (N = 3, n = 4). One-way *ANOVA* with Tukey post test ∗∗∗∗*p* < 0.0001. *C*, representative western blot showing equal amounts of protein lysate from control and PANX1-deficient selected cells generated using two different shRNA constructs (*B* and *D*). Numbers indicate different clones selected from each construct (B1, B2, and D2). Western blotting was performed using the indicated antibodies. GAPDH was used as a loading control. *D*, PANX1 (*left panel*) or β-catenin (*right panel*) bands in the blots described in (*C*) were quantified with Odyssey V3.0 (LI-COR Biosciences). The data represent the mean ± S.E. (*error bars*) of at least three separate biological samples. Statistical analysis conducted using *Student’s t*-test ∗*p* < 0.05. *E*, total RNA was extracted from the specified cells. Quantitative RT-qPCR was conducted to measure human β-catenin mRNA. The amount of mRNA was corrected to YWHAZ as control in the same sample. mRNA in the A375-P cells was set to 1. The data represent the mean ± S.E. (*error bars*), N = 3, n = 3. Statistical analysis conducted using one-way *ANOVA* with Tukey post test ∗∗∗∗*p* < 0.0001. n.s., not significant. *F*, confocal images of PANX1-deficient (PANX1 shRNA) and control (Control shRNA) cells fixed and stained with anti-PANX1 (*yellow*) and anti-β-catenin (*red*) antibodies. DNA was stained with Hoescht (*blue*). Samples incubated only with Alexa flour secondary antibodies were used as control (Secondary only). Scale bar, 5 μm. Data are representative of at least three independent experiments. *G*, MITF bands in blots described in *C* were quantified with Odyssey V3.0 (*left panel*). The amount of MITF, lymphoid enhancer-binding factor 1 (LEF1) and Axin2 mRNA in PANX1-deficient cells (PANX1 shRNA) was corrected to housekeeping gene as control in the same sample. mRNA in the A375-P cells transfected with control shRNA or nontransfected A375-P cells was set to 1. The data represent the mean ± S.E. (*error bars*), N = 3, n = 3. Statistical analysis conducted using one-way *ANOVA* with Tukey post hoc test ∗*p* < 0.05 compared with A375-P and/or Control shRNA. *H*, MTT assay was conducted on PANX1-deficient and control cells. The data represent the mean ± S.E. (*error bars*), N = 3, n = 3. Statistical analysis conducted using one-way *ANOVA* with Tukey post hoc test ∗∗∗∗*p* < 0.0001.

To investigate the effect of PANX1 on β-catenin function in melanoma cells, we generated PANX1 knockout (PANX1 KO) A375-P and A375-MA2 cells using the CRISPR/Cas9 system. A double nicking strategy was employed to reduce the likelihood of off-target effects. In this method, paired guide RNAs with the ability to cleave single strands were used, which resulted in specific double-strand breaks within the *PANX1* gene. Western blotting confirmed the loss of PANX1 expression (Fig. 3*A*). Deletion of the *PANX1* gene from A375-P and A375-MA2 cells caused a substantial reduction in the abundance of β-catenin and its downstream effector Microphthalmia-Associated Transcription Factor (MITF, Fig. 3*A*). Since the growth rate of PANX1 knockout cells in culture was severely reduced and cells were difficult to maintain for experimental procedures, we knocked down PANX1 in A375-P cells using shRNA. The levels of PANX1 mRNA in cells transfected with control shRNA were comparable with those in nontransfected A375-P cells (Fig. 3*B*). In contrast, PANX1 mRNA in cells that were transfected with either of two different shRNA constructs against PANX1 (PANX1shRNA-B and D) was 3.2 ± 0.04-fold lower than control cells (Fig. 3*B*). Similarly, the amount of PANX1 protein was decreased by 4.2 ± 0.02-fold compared with control samples (Fig. 3, *C* and *D*, *left panel*). Analogous to our observations with PANX1 KO cells, the abundance of β-catenin was significantly reduced in PANX1 knockdown melanoma cells (Fig. 3, *C* and *D*, *right panel*). To evaluate whether loss of PANX1 affects the β-catenin mRNA, we performed quantitative RT-qPCR. A375-P cells transfected with shRNA against β-catenin were used as control (Fig. 3*E*). Levels of β-catenin mRNA among nontransfected, control shRNA and PANX1 shRNA transfected A375-P cells were comparable (Fig. 3*E*). These data indicate that PANX1 likely contributes to the posttranscriptional modification and/or stability of β-catenin protein in melanoma cells and does not have a significant impact on β-catenin mRNA levels.

Next, we validated our findings by immunofluorescence analysis in PANX1-deficient melanoma cells. In cells transfected with control shRNA, β-catenin is abundant at the cell membranes where it partially colocalizes with membrane-bound PANX1 (Fig. 3*F*). In contrast, in PANX1-deficient cells, PANX1 is decreased and less abundant at the cell surface and areas of cell–cell contact. In addition, the pattern of β-catenin staining at cell borders is also irregular and disrupted when PANX1 is reduced in melanoma cells (Fig. 3*F*).

MITF is a melanocyte lineage-specific transcription factor that is linked to plasticity of melanoma cells and has key roles in proliferation, migration, and invasiveness of melanoma cells (27, 28). The promoter of human and mouse *Mitf-M*, a specific isotype in melanocytes, contains β-catenin-binding sites (29). To assess the effect of reduced β-catenin on MITF expression in PANX1-deficient and knockout cells, we performed western blotting (Fig. 3, *A* and *C*). MITF protein was significantly reduced in PANX1-deficient melanoma cells (Fig. 3, *C* and *G*). Similarly, qPCR analysis revealed a significant reduction in the mRNA of MITF when PANX1 is knocked down in A375-P cells (Fig. 3*G*). MITF expression is modulated through β-catenin and another key effector in the Wnt pathway called lymphoid enhancer-binding factor 1 (LEF1) in melanoma cells (30, 31, 32). Moreover, MITF can cooperate with LEF1 as a coactivator to enhance its own expression (32). Additionally, *LEF1* has been reported as a Wnt/β-catenin target gene in several cell lines (33, 34, 35), including melanoma (36, 37, 38). LEF1 mRNA was significantly reduced when we knocked down PANX1 in A375-P melanoma cells (Fig. 3*G*). In addition, mRNA levels of Axin2, which is a common β-catenin target gene, were substantially reduced upon PANX1knockdown but did not reach statistical significance (Fig. 3*G*).

Wnt/β-catenin signaling is a major regulator of melanoma proliferation (16, 39, 40). In agreement with this notion, PANX1-deficient A375-P cells showed significantly decreased growth rate compared with control cells starting 2 days after seeding (Fig. 3*H*). These results demonstrate that expression of several key effectors of the Wnt signaling pathway, including β-catenin, is reduced in PANX1-deficent melanoma cells.

### β-Catenin regulates PANX1 expression in melanoma cells

**Figure 4:**
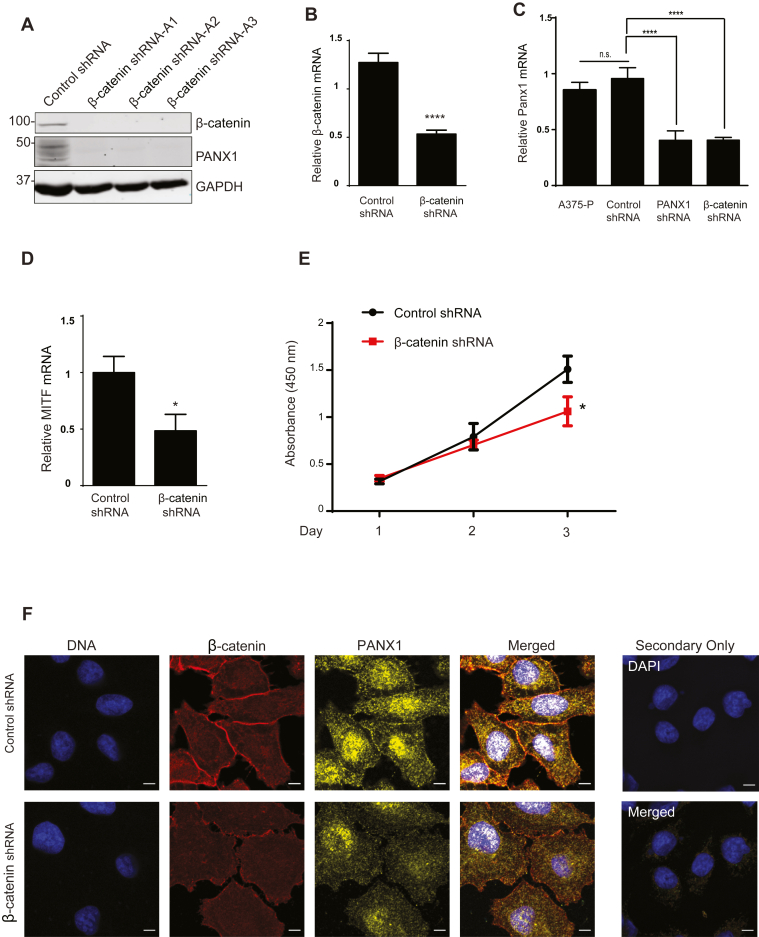
**β-catenin regulates PANX1 expression in melanoma cells.***A*, western blot showing equal amounts of protein lysate from control and β-catenin-deficient cells (clones A1, A2, and A3), probed using the indicated antibodies. GAPDH was used as a loading control. *B*, relative amount of β-catenin mRNA in cells transfected with shRNA against β-catenin was quantified using RT-qPCR and corrected to YWHAZ as control in the same sample. ∗∗∗*p* < 0.0001, *Student’s t*-test. *C*, relative amount of PANX1 mRNA in cells transfected with shRNA against β-catenin or against PANX1 was quantified as explained in Panel *B*. The data represent the mean ± S.E. (*error bars*), N = 3, n = 3. ∗∗∗∗*p* < 0.0001, one-way *ANOVA* with Tukey post hoc test. n.s., not significant. *D*, relative amount of MITF mRNA in cells transfected with β-catenin shRNA or control shRNA. The data represent the mean ± S.E. (*error bars*), N = 3, n = 3. Statistical analysis conducted using *Student’s t*-test ∗*p* < 0.05. *E*, MTT assays were conducted on β-catenin-deficient cells and control cells. The data represent the mean ± S.E. (*error bars*), N = 3, n = 3. Statistical analysis conducted using one-way *ANOVA* with Tukey post hoc test ∗*p* < 0.05. *F*, cells transfected with β-catenin shRNA or control shRNA were fixed and stained with anti-PANX1 (*yellow*) and anti-β-catenin (*red*) antibodies. DNA was stained with Hoescht (*blue*). Scale bar, 5 μm. The data are representative of at least three independent experiments.

The effect of shRNA knockdown (KD) of β-catenin on the expression of PANX1 in melanoma cells was evaluated in A375-P cells. Western blotting and qPCR confirmed reduced β-catenin protein (Fig. 4*A*) and mRNA levels (Fig. 4*B*) after β-catenin shRNA KD, as expected. Interestingly, the levels of both PANX1 protein and mRNA were substantially reduced in β-catenin-deficient melanoma cells (Fig. 4, *A* and *C*). The 1.9-fold decrease in PANX1 mRNA when β-catenin was knocked down was the same as knocking down PANX1 using PANX1 shRNA (Fig. 4*C*). Knocking down β-catenin in melanoma cells also reduced MITF mRNA by 2.8-fold (Fig. 4*D*). The role of Wnt/β-catenin/MITF pathway in melanoma cell proliferation is well established (27, 30). In agreement with this fact, there was a significant reduction in the growth rate of A375-P cells with β-catenin knockdown (Fig. 3*E*). The effect of β-catenin reduction on PANX1 subcellular localization was investigated using immunofluorescence analysis (Fig. 4*F*). Our findings suggest that in β-catenin knocked down A375-P cells, PANX1 is reduced, particularly in its localization at the cell surface and areas of cell–cell contact, and shows a diffuse intracellular distribution (Fig. 4*F*).

### Long-term exposure to PANX1 blockers decreases the abundance of PANX1 and β-catenin in melanoma cells

**Figure 5:**
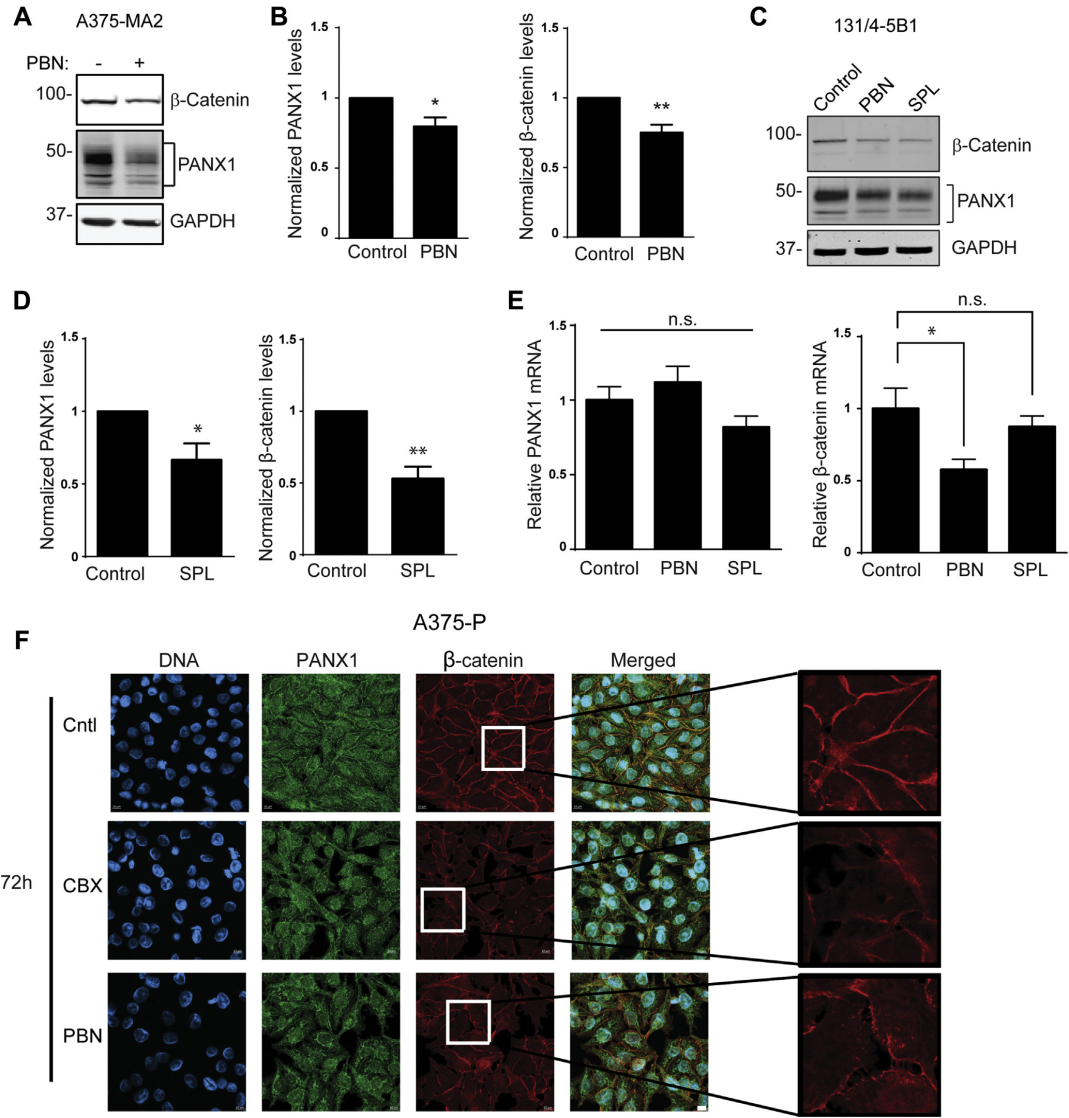
**Long-term exposure to PANX1 blockers decreases the abundance of PANX1 and β-catenin in melanoma cells.***A*, A375-MA2 cells were incubated with 1 mM PBN for 72 h (+). HBSS was used as vehicle control (−). Equal amounts of protein lysate were resolved by SDS-PAGE. Western blotting was performed using indicated antibodies. GAPDH was used as a loading control. *B*, the PANX1 (*left panel*) or β-catenin (*right panel*) bands in the blots described in *A* were quantified with Odyssey V3.0 (LI-COR Biosciences). The data represent the mean ± S.E. (*error bars*), N = 3, n = 3. ∗*p* < 0.05, ∗∗*p* < 0.01, *Student’s t*-test. *C*, 131/4-5B1 cells were incubated with 1 mM PBN or 10 μM SPL for 72 h. Equal amounts of cytoplasmic fraction of cell lysates were resolved by SDS-PAGE. Western blotting was performed using indicated antibodies. GAPDH was used as a loading control. *D*, PANX1 (*left panel*) or β-catenin (*right panel*) bands in blots described in (*C*) were quantified as described for panel *B*. *Student’s t* test ∗*p* < 0.05, ∗∗*p* < 0.01 compared with control cells treated with HBSS as vehicle control. *E*, the relative amount of PANX1 (*middle panel*) or β-catenin (*right panel*) mRNA in PBN- and SPL-treated cells was corrected to housekeeping gene in the same sample. Relative mRNA in cells treated with HBSS as vehicle control was set to 1. The data represent the mean ± S.E. (*error bars*), N = 3, n = 3. Statistical analysis conducted using one-way *ANOVA* with Tukey post hoc test ∗*p* < 0.05. n.s., not significant. *F*, cells treated with 100 μM Carbenoxolones (CBX) or 1 mM Probenecid (PBN) were fixed and stained with anti-PANX1 (*yellow*) and anti-β-catenin (*red*) antibodies. DNA was stained with Hoescht (*blue*). Scale bar, 10 μm. The data are representative of at least three separate experiments.

We previously reported that long-term exposure to PANX1 channel blockers, namely Carbenoxolone (CBX) and Probenecid (PBN), reduces the growth rate of melanoma cells evident after 3 days in culture (11), a phenotype that is also observed in cells with depleted PANX1 protein (Fig. 3*H*). To assess the effect of long-term exposure to PANX1 blockers, we incubated A375-MA2 cells with PBN for 72 h. Quantification revealed that PBN decreases protein levels of both PANX1 (by 20%) and β-catenin (by 30%) (Fig. 5, *A* and *B*). Spironolactone (SPL) was recently shown to specifically block PANX1 (41). Incubation of highly invasive 131/4-5B1 melanoma cells with either PBN or SPL markedly reduced cytoplasmic levels of both PANX1 and β-catenin (Fig. 5, *C* and *D*). SPL caused about 37% ± 11 reduction in PANX1 and 47% ± 8 reduction in β-catenin levels (Fig. 5, *C* and *D*). qPCR showed that neither PBN nor SPL significantly altered PANX1 mRNA (Fig. 5*E*, *left panel*), which suggests that long-term exposure to blockers likely affects PANX1 at the protein level.

Treatment with PBN but not SPL reduced the β-catenin mRNA levels (Fig. 5*E*, *right panel*), suggesting that PBN likely suppresses β-catenin not only through reduction of PANX1 but also through inhibition of β-catenin mRNA transcription. Immunofluorescence analysis of A375-P cells demonstrated that both CBX and PBN reduce PANX1 levels and localization at the cell membrane (Fig. 5*F*) and altered the cell membrane localization of β-catenin (Fig. 5*F*, *insets*) in a manner similar to either PANX1 or β-catenin knockdown cells (Figs. 3*F*, 4*F* and 5*E*). Interestingly, 72 h exposure to CBX and PBN also alters calmodulin subcellular localization in melanoma cells causing more accumulation of calmodulin around nuclear compartments (Fig. S1*B*). Given the association of PANX1 and calmodulin (Fig. 1*E*, Fig. S1*A*), it is likely that depletion of PANX1 alters the subcellular localization of its binding partners, including calmodulin. Overall, our findings suggest that long-term exposure to PANX1 blockers reduces PANX1 and β-catenin proteins and changes their subcellular localization in melanoma cells.

### PANX1-deficient melanoma cells have impaired metabolic activity

**Figure 6:**
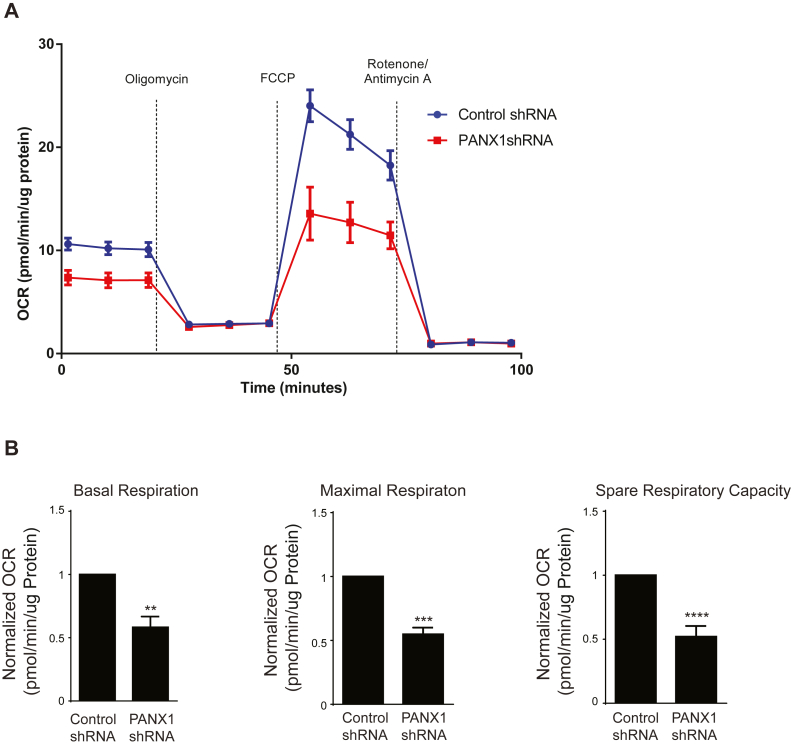
**PANX1-deficient melanoma cells have impaired mitochondrial metabolic activity.***A*, control and PANX-1 shRNA transfected A375-P cells were incubated in XF DMEM at 37 °C, 0% CO_2_ for 1 h prior to Seahorse XF^e^24 extracellular flux analysis. Representative normalized oxygen consumption rate (OCR) during the mitochondrial stress test is shown. *B*, a mitochondrial stress test was used to determine basal respiration, maximal respiration, and spare respiratory capacity. Carbonyl cyanide-4 (trifluoromethoxy) phenylhydrazone (FCCP) was used as electron transport chain uncoupling agent. OCR was normalized to total protein/well and Agilent Seahorse Wave Desktop and XF Cell Mito Stress Test Report Generator software were used for data processing. The data represent the mean ± S.E. (*error bars*), N = 3, n = 2. Statistical analysis conducted using *Student’s t*-test ∗∗*p* < 0.01, ∗∗∗*p* < 0.001, ∗∗∗∗*p* < 0.0001.

The role of Wnt signaling in controlling cancer metabolism is well established (19). The Wnt pathway is implicated in the regulation of bioenergetics in melanoma cells in a β-catenin-dependent manner (42). We hypothesized that reduction of PANX1 and consequently β-catenin would alter the metabolic profile of melanoma cells. To investigate the real-time effect of PANX1 knockdown on mitochondrial activity, oxygen consumption rate (OCR) of cells transfected with shRNA against PANX1 was measured using a mitochondrial stress test with a Seahorse XF analyzer (Fig. 6*A*). PANX1-deficient cells exhibited significantly lower basal respiration than cells transfected with control shRNA (Fig. 6*B*, *left panel*). Furthermore, following the injection of carbonyl cyanide-4 (trifluoromethoxy) phenylhydrazone (FCCP), an electron transport chain uncoupling agent, maximal respiration (Fig. 6*B*, *middle panel*) and spare respiratory capacity (Fig. 6*B*, *right panel*) were also decreased in PANX1 knockdown cells. These results suggest that PANX1-deficient cells exhibit suppressed mitochondrial metabolism, which may explain reduced proliferation and the previously reported reduction in the migration of these cells (11).

## Discussion

Since the discovery of PANX1 by Panchin *et al.* in 2000 (43), a growing body of evidence is showing key roles for PANX1 in the regulation of cancer progression (44). Originally considered as a channel-forming protein at the cell surface to facilitate the release of ATP (5), PANX1 has been predominantly studied as a cell-membrane-associated channel protein, and potential roles of intracellular PANX1 are less characterized. However, recent findings indicate a signaling role for intracellular PANX1 through its interaction with a variety of cytoplasmic proteins and signaling molecules, such as collapsing response mediator protein 2 (45), actin and actin-interacting protein Arp3 (46), and inflammasome components including caspase-1, XIAP, and ASC (47, 48). Here we demonstrate that PANX1 binds directly to the Wnt pathway transcription factor, β-catenin. Further, we propose that PANX1 likely contributes to the stability of β-catenin protein in melanoma cells through modulation of β-catenin protein levels and regulates β-catenin transcriptional activity in these cells. Our findings provide a mechanistic insight into the role of PANX1 in regulation of cellular processes, such as proliferation, migration, and invasion during melanoma progression.

The evolutionarily conserved Wnt/β-catenin pathway controls diverse and varied processes contributing to cellular functions and tissue homeostasis (49). The tight control over the functions of Wnt/β-catenin pathway is highly dependent on its interactome (50). For example, several actin-related molecules, such as adenomatous polyposis coli (APC), directly bind to β-catenin and modulate its function (51). Our study indicates that PANX1 associates with β-catenin in the melanoma cell milieu. Immunofluorescence imaging reveals areas of PANX1 and β-catenin colocalization at cell membrane as well as intracellularly (Figs. 3*F* and 4*F*). In-depth analysis of possible intracellular interactions between PANX1 and β-catenin will provide better understanding of PANX1-mediated signaling. An important finding in our study is a direct interaction between PANX1 and β-catenin. Binding to β-catenin is mediated through the C-terminal region of PANX1, which has been shown to interact with actin and actin-interacting protein Arp3 (46, 52). Also, PANX1 binds to the N-terminal region of β-catenin, which harbors almost all the carcinogenic mutations that have been identified in β-catenin (53). Thus, binding of PANX1 to the N-terminal portion of β-catenin may have critical impact on β-catenin structure and function. Overall, our findings establish PANX1 among a plethora of β-catenin-binding partners that either directly affect its transcriptional activity or allow its direct cross talk with other signaling effector molecules.

In a spontaneous melanoma mouse model with melanocyte-specific Pten-inactivation and the Braf^V600E^-activating mutation, loss of β-catenin inhibits melanoma formation and suppresses the ability of melanoma tumor to metastasize to distant organs (54). Also, active β-catenin signaling inside melanoma tumors dominantly excludes T-cell infiltration into the melanoma microenvironment, thus preventing antitumor immune response (18). We have shown that the abundance of β-catenin decreases substantially upon PANX1 depletion from both mouse (12) and human melanoma cells (Fig. 3). In addition, two Wnt/β-catenin target genes were significantly reduced, namely *MITF* and *LEF1*, as well as *Axin2*, a common β-catenin target gene, was reduced upon PANX1 knock-down by 2.4, 2.2, and 3.4-fold, respectively (Fig. 3). Both MITF and LEF1 have been implicated in melanoma survival and resistance (28, 55). Furthermore, PANX1 inhibition is associated with significant reduction in tumor growth (11, 12) and distant metastasis tested in our *ex vivo* model of chick chorioallantoic membrane (chick –CAM) (12). Thus, PANX1 likely regulates formation and metastasis of melanoma tumor in cross talk with Wnt/β-catenin pathway.

Stabilization of β-catenin (*e.g.*, through phosphorylation on exon 3) is an alternative way for increasing its activity, which can happen even without signaling inputs from Wnt ligands (56, 57, 58). Our data indicate that loss of PANX1 does not change β-catenin mRNA, suggesting that PANX1 may alter the stability of β-catenin at the protein level. Additionally, two specific PANX1 blockers tested in our study significantly reduce the abundance of both PANX1 and β-catenin proteins in melanoma cells. In agreement with that notion, one recent study has shown that the PANX1 blocker, PBN, reduces PANX1 and β-catenin levels in breast cancer cells and suppresses their invasiveness and metastatic potential (59). These findings support our previously published data showing that PANX1 blockers reduce invasiveness and metastatic capacity of melanoma cells (11, 12). Together, we identified the FDA-approved PANX1 inhibitors as repurposed drugs that could potentially be used in clinical settings for diseases with altered PANX1-mediated β-catenin signaling.

In the canonical Wnt signaling, pathway is activated mainly *via* Wnt3a binding to the FZD/LRP receptor, leading to disruption in the “destruction complex” and consequently β-catenin translocation to the nucleus, which activates transcription of Wnt target genes (13). In contrast, when Wnt signaling is OFF, the destruction complex is stabilized, which contains several interactor molecules that contribute to β-catenin posttranslational modification (mainly phosphorylation and ubiquitin-mediated degradation), thereby attenuating transcription (60). Changes in subcellular localization of β-catenin are an important step in the regulation of transcriptional activity (61). Notably, our findings show that β-catenin does not associate with the cell membrane in the absence of PANX1 (Fig. 3*F*). Dissociation of β-catenin from the cell border is likely to increase the exposure of cytoplasmic β-catenin to degradation, which is an important regulatory mechanism of Wnt/β-catenin in cancer (50). In melanoma cells, β-catenin increases proliferation and growth through interaction with Brn2 transcription factor (39). Similar to our observation with PANX1-deficient cells, knocking down β-catenin significantly decreased the growth rate of melanoma cells in our experimental setting (Fig. 4), confirming the key role of β-catenin in melanoma cell homeostasis.

The association of PANX1 with its binding partners is modulated through signaling inputs among which Ca^2+^ is of great importance. Our data established that the interaction of endogenous PANX1 with calmodulin, a master regulator of Ca^2+^ signaling in eukaryotic cells, is regulated through Ca^2+^. Therefore, it is reasonable to postulate that Ca^2+^ alters the PANX1 interactome, regulating the PANX1-mediated signaling network. Recent studies have shown the role of noncanonical Wnt5A-mediated signaling in melanoma cellular processes through calcium-dependent enzymes (62). Thus, depending on the context, both canonical and noncanonical Wnt signaling contributes to melanoma proliferation, migration, and invasion (40). Our data showing the interaction of PANX1 with β-catenin and calmodulin provide a hint to a potential role for PANX1 in both canonical Wnt3a/β-catenin-dependent and noncanonical Wnt5a/Ca^2+^ signaling.

In a context-dependent cross talk with Ca^2+^ signaling, Wnt/β-catenin pathway increases mitochondrial networking (63) and regulates the bioenergetic potential as well as metabolic profile of several cell types (63, 64), including melanoma cells (42, 63). In addition, a majority of β-catenin-binding proteins in PTEN^wt^ A375-P melanoma cells are involved in metabolic processes, which are vastly altered by Wnt3A-mediated signaling (42). Our observation of the novel interaction between PANX1 and β-catenin prompted us to investigate the mitochondrial metabolic profile of PANX1-deficent cells. Our data revealed that reducing PANX1 in melanoma cells suppresses their mitochondrial metabolism (Fig. 6). It is likely that PANX1 regulates the metabolic profile of melanoma cells through modulation of Wnt/β-catenin signaling.

In this paper, we document a previously undescribed direct association of PANX1 with a key transcription factor of the Wnt signaling, β-catenin. We have shown that PANX1 modulates β-catenin’s stability and transcriptional activity. Our findings indicate an indirect way to modulate β-catenin at the protein level through FDA-approved repurposed drugs, such as PBN and SPL that can potentially be used in other contexts and diseases. These observations expand our understanding of both PANX1 and β-catenin regulation and establish a novel cross talk between PANX1 and the Wnt signaling pathway that can potentially be target for new therapeutic interventions in patients with malignant melanoma.

## Experimental procedures

### *In silico* analysis of correlation between PANX1 and β-catenin protein expression in melanoma and breast cancer tumors

PANX1 mRNA expression z-scores (RNA Seq V2 RSEM) and CTNNB1 mRNA expression z-scores (RNA Seq V2 RSEM) were generated using data in cBioPortal.org from the Skin Cutaneous Melanoma and Breast Invasive Carcinoma Cohorts generated by the TCGA Research Network (http://cancergenome.nih.gov).

### Cell lines and culture conditions

Human melanoma cells lines, A375P (ATCC CRL-3224), A375-MA2 (ATCC CRL-3223), A2058 (ATCC CRL-11147) were obtained from ATCC while cell line 131/4-5B1 was a gift from Dr Kerbel (65) and cultured in Dulbecco’s Modified Eagle Medium 1X (DMEM 1X) containing 4.5 g/l D-glucose, L-glutamine, 110 mg/l sodium pyruvate, 10% fetal bovine serum (FBS, Invitrogen), 100 units/ml penicillin, and 0.1 mg/ml streptomycin. All cells were incubated at 37 °C at 5% CO_2_. Trypsin (0.25%, 1 mM EDTA 1X; Life Technologies) was used to dissociate cells from culture dishes.

### Protein extraction and immunoblotting

Protein lysates were extracted with: 1% Triton X-100, 150 mM NaCl, 10 mM Tris, 1 mM EDTA, 1 mM EGTA, 0.5% NP-40, or a RIPA buffer (50 mM Tris-HCl pH 8.0, 150 mM NaCl, 1% NP-40 (Igepal), 0.5% sodium deoxycholate). Each buffer contained 1 mM sodium fluoride, 1 mM sodium orthovanadate, and half of a tablet of complete-mini EDTA-free protease inhibitor (Roche). Protein was quantified by bicinchoninic acid (BCA) assay (Thermo Scientific). Protein lysates (40 μg) were separated by 10% SDS-PAGE and transferred onto a nitrocellulose membrane using an iBlotTM System (Invitrogen). Membranes were blocked with 3% bovine serum albumin (BSA) with 0.05% Tween-20 in 1X phosphate buffer saline (PBS), and incubated with anti-human PANX1 antibody (1:1000; PANX1 CT-412; 0.35 μg/μl) (66), anti-human β-catenin (BD Transduction Lab # 610154), MITF (abcam # ab20663). Development and characterization of the anti-calmodulin monoclonal antibody have been described (67) by Sacks at NIH. Loading controls were done with an anti-GAPDH antibody (1:1000; Millipore Cat# MAB374). For detection, IRDye -800CW and -680RD (Life TechnologiesTM) were used as secondary antibodies at 1:10,000 dilutions and imaged using a Li-Cor Odyssey infrared imaging system (Li-Cor). Western blot quantification and analysis were conducted using Image Studio Lite (LI-COR Biosciences). Subcellular fractionation was conducted using NE-PER Nuclear and Cytoplasmic Extraction Kit (ThermoFisher, # 78833) following the manufacturer's instruction. Samples were resolved with SDS-PAGE and subjected to western blotting as described above.

### PANX1 inhibitors

Carbenoxolone disodium salt (≥98%; Sigma Aldrich) and water-soluble Probenecid (77 mg/ml; Invitrogen) were dissolved in Hanks’s Balanced Salt Solution (HBSS 1X, Life Technologies; calcium chloride, magnesium chloride, magnesium sulfate) to develop stock concentrations of each compound. Spironolactone was purchased from Selleckchem (#52-01-7).

### Generation of Crispr/Cas9 knock out cells

PANX1 knockout cells were generated using CRISPR/Cas9 D10A following Ran *et al.*, (68) protocol. Briefly, cells were transfected with 1 μg each of pSpCas9n(BB)-2A-Puro (PX462) V2.0 and pSpCas9n(BB)-2A-GFP (PX461) (addgene.org) containing guide RNA sequences for human PANX1 in a 6-well plate. PANX1 gRNAs were designed with http://tools.genome-engineering.org (sequences GTTCTCGGATTTCTTGCTGA and CTCCGTGGCCAGTTGAGCGA). Twenty-four hours post transfection, cells were selected with 1 μg/ml Puromycin for 72 h. Following selection, cells were screened for PANX1 levels by western blot. Plasmids were a gift from Feng Zhang (Addgene plasmid #48140 and #62987).

### shRNA knockdown of PANX1 and β-catenin

A375-P cells were transfected with two constructs (PANX1 shRNA-B and PANX1 shRNA-D) from Origene PANX1 human 29-mer shRNA kit in pRS vector (#TR302694) (sequence: 5’-CGCAATGCTACTCCTGACAAACCTTGGCATGTCAAGAGCATGCCAAGGTTTGTCAGGAGTAGCATTGTT-3’) plus a pLKO.1 sh β-catenin.1248 (CCGGAGGTGCTATCTGTCTGCTCTACTCGAGTAGAGCAGACAGATAGCACCTTTTTT) (#19761) GFP shRNA cassette (5’GCCCGCAAGCTGACCCTGAAGTTCATTCAAGAGATGAACTTCAGGGTCAGCTTGCTTTTT-3’) from Addgene (#30323) as a control. Control shRNA transfected cells were evaluated for PANX1 and β-catenin expression compared with nontransfected cells. Single cell colony of PANX shRNA-expressing cells from two constructs (B and D) were selected with puromycin and examined for PANX1 knockdown (KD). Stable knockdown samples showed 80 to 90% reduction in PANX1 expression. Cells were maintained under puromycin selection pressure and periodically examined for effective PANX1 knockdown by western blot. Similarly, single cell colony selected β-catenin shRNA transfected cells were examined for 80 to 90% reduction in β-catenin expression. Cells were maintained under puromycin selection pressure and periodically examined for effective β-catenin knockdown by western blotting. Experiments were conducted after verifying at least a 70 to 85% knockdown of PANX1 and β-catenin protein levels by western blotting.

### Transcription and translation (T_N_T) product production and binding analysis

[^35^S]Methionine-labeled T_N_T products were synthesized using the T_N_T quick coupled transcription/translation system (Promega) essentially as described previously (69). Briefly, 1 μg of each plasmid was incubated with 40 μl of T_N_T Quick Master mix and 20 μCi of [^35^S]methionine (PerkinElmer Life Sciences) for 90 min at 30 °C. T_N_T products were confirmed by SDS-PAGE and autoradiography before being used in pull-down assays. To identify the region of β-catenin that binds to PANX1, T_N_T products of PANX1 were incubated with maltose-binding protein (MBP)-β-catenin (MBP alone was used as control) for 3 h at 4 °C. Complexes were washed five times with wash buffer containing 50 mm Tris-HCl, pH 7.4, 150 mm NaCl, and 1% Triton X-100 and separated by SDS-PAGE. The gels were dried, and autoradiography was performed. To identify the region of PANX1 that binds to β-catenin, portions of PANX1 were expressed with TNT. Radiolabeled products were incubated with MBP-β-catenin and processed as described above.

### Protein purification and pull-down assay

pcDNA-PANX1(HCVp) was purchased from Addgen (Plasmid#87698). pcDNA3-myc-PANX1-C was used to make PCR product of PANX1-C with pcDNA-PANX1 as template and 5’CGGGATCCGCTCCCGTGGTTGTCTACACGCTG-3’ as positive primer, 5’-CCGGAATTCTCTAGATCAGCAAGAAGAATCCAGAAGTCTC-3’ as negative primer. The PCR product was cut with BamH I and Xba I and inserted into pcDNA3-myc at BamH I and Xba I site.

pPET28a-TEV-hβ-Catenin-N (amino acids 1–137) (Plasmid#17203), pPET28a-TEV-hβ-Catenin-R1 (M and C domains, amino acids 138–781) (Plasmid#17200) and pPET28a-hβ-Catenin-C (amino acids 666–781) (Plasmid#17204) were purchased from Addgene. His-tagged proteins were expressed in BL21 strain of *E. Coli* and purified with Talon Metal Affinity Resin according to the manufacturer’s protocol.

For binding assays, pcDNA3-myc-PANX1-C was transfected into HEK293 cells. Cells were harvested 72 h post transfection using Buffer A (50 mM Tris, pH 7.4, 150 mM NaCl, 1% Triton-X100). Cells were lysed using sonication and spun down. The supernatants were precleared with Talon beads for 1 h at 4 °C. Equal amounts of protein lysate were incubated with His-tagged β-catenin-N, -R1 or -C attached to Talon beads, or empty Talon beads alone (negative control). Samples were rotated at 4 °C for 3 h and washed 5x with Buffer A. Then, samples were resuspended in 30 μl SDS-Sample buffer. Proteins were separated by 15% SDS-PAGE. The gel was cut below 25 kDa. The upper part was stained with Coomassie blue. The lower part was transferred to PVDF membranes and probed with anti-myc antibody (Millipore anti-Myc-Tag Cat# 06-549).

### Immunoprecipitation

131/4-5B1 or A375-P cells were plated in 10-cm dishes to reach 80% confluence. The following day, the cells were washed with ice-cold PBS and lysed with 500 μl of Buffer A (50 mM Tris-HCl, pH 7.4, 150 mM NaCl, and 1% Triton X-100) with either 1 mM CaCl2 (+) or EGTA(−) supplemented with complete protease and phosphatase inhibitors (Roche). Lysates were subjected to two rounds of sonication for 10 s each, and insoluble material was precipitated by centrifugation at 13,000*g* for 10 min at 4 °C. Supernatants were precleared with protein A-Sepharose beads for 1 h. Equal amounts of protein lysate were incubated with protein A-Sepharose beads and anti-PANX1 polyclonal antibodies or anti-β-catenin monoclonal antibody for overnight at 4 °C. Rabbit IgG and mouse IgG were used as controls for polyclonal and monoclonal antibody immunoprecipitations, respectively. Samples were washed five times with Buffer A, resolved by SDS-PAGE, and Quick Western detection kit (LiCore #926-69100) was used to detect the protein processed by western blotting.

### MTT assay

Cells were cultured at 1000/well onto a 96-well plate. Cell Proliferation Reagent WST-1 (Sigma Aldrich) was used to assess the growth rate of cells according to manufacturer’s instructions. Measurements at 450 nm and at a 690 nm were taken on an Epoch microplate spectrometer (Biotek).

### Quantitative RT-qPCR

To measure mRNA, cells were cultured for 24 h. Then total RNA was isolated from the cells using an RNA isolation kit (Qiagen). In total, 250 ng of RNA was reverse transcribed to cDNA using a high-capacity cDNA reverse transcriptase kit (Applied Biosystems#4374966) according to the manufacturer’s instructions. RT-qPCR was performed using SYBR Green PCR Master Mix (BioRad#1725274) and 200 nM forward and reverse primers. The primers used human PANX1: 5’- AACCGTGCAATTAAGGCTG -3’ (forward); and 5’- GGCTTTCAGATACCTCCCAC -3’ (reverse); β-catenin: 5’- AAAATGGCAGTGCGTTTAG -3’ (forward); and 5’- TTTGAAGGCAGTCTGTCGTA -3’ (reverse); MITF: 5’- CCTTCTCTTTGCCAGTCCATCT -3’ (forward); and 5’- GGACATGCAAGCTCAGGACT -3’ (reverse); and Axin2: 5’-GAGTGGACTTGTGCCGACTTCA-3’ (forward); 5’- GGTGGCTGGTGCAAAGACATAG-3’ (reverse); and Lef1: 5’- CCTGGTCCCCACACAACTG-3’ (forward); and 5’- GGCTCCTGCTCCTTTCTCTG-3’ (reverse); RT-qPCR enzyme activation was initiated for 10 min at 95 °C and then amplified for 40 cycles of a two-step PCR (15 s at 95 °C and 1 min at 60 °C). All samples were assayed at least in duplicate, and YWHAZ was used as reference control. The results were analyzed using the ΔΔCT method.

### Immunofluorescence microscopy

Cells were grown on glass coverslips and were fixed 72 h posttransfection using ice-cold 8:2 methanol:acetone for 15 min at 4 °C and blocked with 2% BSA-PBS. Coverslips were incubated with anti-human PANX1 antibody (1:500; PANX1 CT-412; 0.35 μg/μl) and anti-mouse β-catenin (1:450 BD Biosciences# 610153). Nuclei was stained with Hoechst 33342 (1:1000), Alexa Fluor 647 anti-mouse and 555 goat anti-rabbit IgG (2 mg/ml, 1:500) were used as secondaries, and mounted using Airvol (Mowiol 4-88; Sigma Aldrich) prior to imaging. Immunofluorescence images were obtained using a Zeiss LSM 800 Confocal Microscope with a Plan-Apochromat 63x/1.40 Oil DIC objective (Carl Zeiss).

### Mitochondrial stress test

Control shRNA and PANX-1 shRNA malignant melanoma cells were seeded at a density of 25,000 to 30,000 cells/well in an XFe24 cell culture microplate (#102340-100; Agilent) pretreated with 0.01% Poly-L-Lysine (Millipore #A-005-C) 1 to 2 days prior to running the assay. On the day of the assay, cell culture medium was replaced with Seahorse XF DMEM medium, pH 7.4 (#103575-100; Agilent) supplemented with 10 and 25 mM glucose (#103577-100; Agilent), 4 mM glutamine (#103579-100; Agilent), and 1 mM pyruvate (#103578-100; Agilent). Cells were incubated in XF DMEM for one h at 37 °C, 0% CO_2_ prior to running the assay. A mitochondrial stress test kit (#103709-100; Agilent) was used to determine basal respiration, maximal respiration, and spare respiratory capacity following the manufacturer’s instructions. OCR was normalized to total protein/well using a DC protein assay (#500-0116; Bio-Rad) or a bicinchoninic acid (BCA) assay (Thermo Scientific), and Agilent Seahorse Wave Desktop and XF Cell Mito Stress Test Report Generator software were used for data processing.

### Statistical analyses

All data are representative of at least three independent experiments conducted with three technical replicates unless otherwise mentioned in the figure legends. Statistical analyses were performed using GraphPad Prism software (version 8.0). Error bars indicate mean ± standard error mean. The details of statistical analysis are provided in figure legends.

## Data availability

All the data described in the study is contained within the article. Raw data available upon request (Dr Silvia Penuela, Western University, spenuela@uwo.ca).

## Supporting information

This article contains supporting information.

## Conflict of interest

The authors declare no conflict of interest of any kind with the contents of this article.

## References

[1] Penuela S., Bhalla R., Gong X.Q., Cowan K.N., Celetti S.J., Cowan B.J., Bai D., Shao Q., Laird D.W. (2007). Pannexin 1 and pannexin 3 are glycoproteins that exhibit many distinct characteristics from the connexin family of gap junction proteins. J. Cell Sci..

[2] Sosinsky G.E., Boassa D., Dermietzel R., Duffy H.S., Laird D.W., MacVicar B., Naus C.C., Penuela S., Scemes E., Spray D.C., Thompson R.J., Zhao H.B., Dahl G. (2011). Pannexin channels are not gap junction hemichannels. Channels (Austin).

[3] Boassa D., Ambrosi C., Qiu F., Dahl G., Gaietta G., Sosinsky G. (2007). Pannexin1 channels contain a glycosylation site that targets the hexamer to the plasma membrane. J. Biol. Chem..

[4] Scemes E., Spray D.C., Meda P. (2009). Connexins, pannexins, innexins: Novel roles of “hemi-channels”. Pflugers Arch..

[5] Bao L., Locovei S., Dahl G. (2004). Pannexin membrane channels are mechanosensitive conduits for ATP. FEBS Lett..

[6] D'Hondt C., Ponsaerts R., De Smedt H., Vinken M., De Vuyst E., De Bock M., Wang N., Rogiers V., Leybaert L., Himpens B., Bultynck G. (2011). Pannexin channels in ATP release and beyond: An unexpected rendezvous at the endoplasmic reticulum. Cell. Signal..

[7] Vanden Abeele F., Bidaux G., Gordienko D., Beck B., Panchin Y.V., Baranova A.V., Ivanov D.V., Skryma R., Prevarskaya N. (2006). Functional implications of calcium permeability of the channel formed by pannexin 1. J. Cell Biol..

[8] Penuela S., Harland L., Simek J., Laird D.W. (2014). Pannexin channels and their links to human disease. Biochem. J..

[9] Jiang J.X., Penuela S. (2016). Connexin and pannexin channels in cancer. BMC Cell Biol..

[10] Siegel R.L., Miller K.D., Jemal A. (2019). Cancer statistics, 2019. CA Cancer J. Clin..

[11] Freeman T.J., Sayedyahossein S., Johnston D., Sanchez-Pupo R.E., O'Donnell B., Huang K., Lakhani Z., Nouri-Nejad D., Barr K.J., Harland L., Latosinsky S., Grant A., Dagnino L., Penuela S. (2019). Inhibition of pannexin 1 reduces the tumorigenic properties of human melanoma cells. Cancers (Basel).

[12] Penuela S., Gyenis L., Ablack A., Churko J.M., Berger A.C., Litchfield D.W., Lewis J.D., Laird D.W. (2012). Loss of pannexin 1 attenuates melanoma progression by reversion to a melanocytic phenotype. J. Biol. Chem..

[13] Xue G., Romano E., Massi D., Mandala M. (2016). Wnt/beta-catenin signaling in melanoma: Preclinical rationale and novel therapeutic insights. Cancer Treat. Rev..

[14] Louphrasitthiphol P., Chauhan J., Goding C.R. (2019). ABCB5 is activated by MITF and beta-catenin and is associated with melanoma differentiation. Pigment Cell Melanoma Res..

[15] Lin Y., Wang F., Xing Q., Guo F., Wang M., Li Y. (2018). The biological effect and mechanism of the Wnt/beta-catenin signaling pathway on malignant melanoma A375 cells. Exp. Ther. Med..

[16] Fan G., Ye D., Zhu S., Xi J., Guo X., Qiao J., Wu Y., Jia W., Wang G., Fan G., Kang J. (2017). RTL1 promotes melanoma proliferation by regulating Wnt/beta-catenin signalling. Oncotarget.

[17] Tucci M., Passarelli A., Mannavola F., Felici C., Stucci L.S., Cives M., Silvestris F. (2019). Immune system evasion as hallmark of melanoma progression: The role of dendritic cells. Front. Oncol..

[18] Spranger S., Bao R., Gajewski T.F. (2015). Melanoma-intrinsic beta-catenin signalling prevents anti-tumour immunity. Nature.

[19] Sherwood V. (2015). WNT signaling: An emerging mediator of cancer cell metabolism?. Mol. Cell. Biol..

[20] Furlow P.W., Zhang S., Soong T.D., Halberg N., Goodarzi H., Mangrum C., Wu Y.G., Elemento O., Tavazoie S.F. (2015). Mechanosensitive pannexin-1 channels mediate microvascular metastatic cell survival. Nat. Cell Biol..

[21] Khramtsov A.I., Khramtsova G.F., Tretiakova M., Huo D., Olopade O.I., Goss K.H. (2010). Wnt/beta-catenin pathway activation is enriched in basal-like breast cancers and predicts poor outcome. Am. J. Pathol..

[22] Vincent K.M., Postovit L.M. (2017). Investigating the utility of human melanoma cell lines as tumour models. Oncotarget.

[23] Berchtold M.W., Villalobo A. (2014). The many faces of calmodulin in cell proliferation, programmed cell death, autophagy, and cancer. Biochim. Biophys. Acta.

[24] Chen Y., Zhou Y., Lin X., Wong H.C., Xu Q., Jiang J., Wang S., Lurtz M.M., Louis C.F., Veenstra R.D., Yang J.J. (2011). Molecular interaction and functional regulation of connexin50 gap junctions by calmodulin. Biochem. J..

[25] Siu R.C., Smirnova E., Brown C.A., Zoidl C., Spray D.C., Donaldson L.W., Zoidl G. (2016). Structural and functional consequences of connexin 36 (Cx36) interaction with calmodulin. Front. Mol. Neurosci..

[26] Zou J., Salarian M., Chen Y., Veenstra R., Louis C.F., Yang J.J. (2014). Gap junction regulation by calmodulin. FEBS Lett..

[27] Carreira S., Goodall J., Denat L., Rodriguez M., Nuciforo P., Hoek K.S., Testori A., Larue L., Goding C.R. (2006). Mitf regulation of Dia1 controls melanoma proliferation and invasiveness. Genes Dev..

[28] Leclerc J., Ballotti R., Bertolotto C. (2017). Pathways from senescence to melanoma: Focus on MITF sumoylation. Oncogene.

[29] Larue L., Delmas V. (2006). The WNT/beta-catenin pathway in melanoma. Front. Biosci..

[30] Hartman M.L., Czyz M. (2015). MITF in melanoma: Mechanisms behind its expression and activity. Cell. Mol. Life Sci..

[31] Eichhoff O.M., Weeraratna A., Zipser M.C., Denat L., Widmer D.S., Xu M., Kriegl L., Kirchner T., Larue L., Dummer R., Hoek K.S. (2011). Differential LEF1 and TCF4 expression is involved in melanoma cell phenotype switching. Pigment Cell Melanoma Res..

[32] Saito H., Yasumoto K., Takeda K., Takahashi K., Fukuzaki A., Orikasa S., Shibahara S. (2002). Melanocyte-specific microphthalmia-associated transcription factor isoform activates its own gene promoter through physical interaction with lymphoid-enhancing factor 1. J. Biol. Chem..

[33] Wohrle S., Wallmen B., Hecht A. (2007). Differential control of Wnt target genes involves epigenetic mechanisms and selective promoter occupancy by T-cell factors. Mol. Cell Biol..

[34] Planutiene M., Planutis K., Holcombe R.F. (2011). Lymphoid enhancer-binding factor 1, a representative of vertebrate-specific Lef1/Tcf1 sub-family, is a Wnt-beta-catenin pathway target gene in human endothelial cells which regulates matrix metalloproteinase-2 expression and promotes endothelial cell invasion. Vasc. Cell.

[35] Eastman Q., Grosschedl R. (1999). Regulation of LEF-1/TCF transcription factors by Wnt and other signals. Curr. Opin. Cell Biol..

[36] Lucero O.M., Dawson D.W., Moon R.T., Chien A.J. (2010). A re-evaluation of the “oncogenic” nature of Wnt/beta-catenin signaling in melanoma and other cancers. Curr. Oncol. Rep..

[37] Bellei B., Pitisci A., Catricala C., Larue L., Picardo M. (2011). Wnt/beta-catenin signaling is stimulated by alpha-melanocyte-stimulating hormone in melanoma and melanocyte cells: Implication in cell differentiation. Pigment Cell Melanoma Res..

[38] Shah K.V., Chien A.J., Yee C., Moon R.T. (2008). CTLA-4 is a direct target of Wnt/beta-catenin signaling and is expressed in human melanoma tumors. J. Invest. Dermatol..

[39] Goodall J., Martinozzi S., Dexter T.J., Champeval D., Carreira S., Larue L., Goding C.R. (2004). Brn-2 expression controls melanoma proliferation and is directly regulated by beta-catenin. Mol. Cell. Biol..

[40] Kaur A., Webster M.R., Weeraratna A.T. (2016). In the Wnt-er of life: Wnt signalling in melanoma and ageing. Br. J. Cancer.

[41] Good M.E., Chiu Y.H., Poon I.K.H., Medina C.B., Butcher J.T., Mendu S.K., DeLalio L.J., Lohman A.W., Leitinger N., Barrett E., Lorenz U.M., Desai B.N., Jaffe I.Z., Bayliss D.A., Isakson B.E. (2018). Pannexin 1 channels as an unexpected new target of the anti-hypertensive drug spironolactone. Circ. Res..

[42] Brown K., Yang P., Salvador D., Kulikauskas R., Ruohola-Baker H., Robitaille A.M., Chien A.J., Moon R.T., Sherwood V. (2017). WNT/beta-catenin signaling regulates mitochondrial activity to alter the oncogenic potential of melanoma in a PTEN-dependent manner. Oncogene.

[43] Panchin Y., Kelmanson I., Matz M., Lukyanov K., Usman N., Lukyanov S. (2000). A ubiquitous family of putative gap junction molecules. Curr. Biol..

[44] Graham S.V., Jiang J.X., Mesnil M. (2018). Connexins and pannexins: Important players in tumorigenesis, metastasis and potential therapeutics. Int. J. Mol. Sci..

[45] Xu X., Wicki-Stordeur L.E., Sanchez-Arias J.C., Liu M., Weaver M.S., Choi C.S.W., Swayne L.A. (2018). Probenecid disrupts a novel pannexin 1-collapsin response mediator protein 2 interaction and increases microtubule stability. Front. Cell Neurosci..

[46] Bhalla-Gehi R., Penuela S., Churko J.M., Shao Q., Laird D.W. (2010). Pannexin1 and pannexin3 delivery, cell surface dynamics, and cytoskeletal interactions. J. Biol. Chem..

[47] Silverman W.R., Vaccari J.P.D.R., Locovei S., Qiu F., Carlsson S.K., Scemes E., Keane R.W., Dahl G. (2009). The pannexin 1 channel activates the inflammasome in neurons and astrocytes. J. Biol. Chem..

[48] Wang H., Xing Y., Mao L., Luo Y., Kang L., Meng G. (2013). Pannexin-1 influences peritoneal cavity cell population but is not involved in NLRP3 inflammasome activation. Protein Cell.

[49] Valenta T., Hausmann G., Basler K. (2012). The many faces and functions of beta-catenin. EMBO J..

[50] Shang S., Hua F., Hu Z.W. (2017). The regulation of beta-catenin activity and function in cancer: Therapeutic opportunities. Oncotarget.

[51] Akiyama T., Kawasaki Y. (2006). Wnt signalling and the actin cytoskeleton. Oncogene.

[52] Wicki-Stordeur L.E., Boyce A.K.J., Swayne L.A. (2013). Analysis of a pannexin 2-pannexin 1 chimeric protein supports divergent roles for pannexin C-termini in cellular localization. Cell Commun. Adhes..

[53] Dar M.S., Singh P., Mir R.A., Dar M.J. (2017). Beta-catenin N-terminal domain: An enigmatic region prone to cancer causing mutations. Mutat. Res..

[54] Damsky W.E., Curley D.P., Santhanakrishnan M., Rosenbaum L.E., Platt J.T., Gould Rothberg B.E., Taketo M.M., Dankort D., Rimm D.L., McMahon M., Bosenberg M. (2011). Beta-catenin signaling controls metastasis in Braf-activated Pten-deficient melanomas. Cancer Cell.

[55] Hugo W., Shi H., Sun L., Piva M., Song C., Kong X., Moriceau G., Hong A., Dahlman K.B., Johnson D.B., Sosman J.A., Ribas A., Lo R.S. (2015). Non-genomic and immune evolution of melanoma acquiring MAPKi resistance. Cell.

[56] Omholt K., Platz A., Ringborg U., Hansson J. (2001). Cytoplasmic and nuclear accumulation of beta-catenin is rarely caused by CTNNB1 exon 3 mutations in cutaneous malignant melanoma. Int. J. Cancer.

[57] Demunter A., Libbrecht L., Degreef H., De Wolf-Peeters C., van den Oord J.J. (2002). Loss of membranous expression of beta-catenin is associated with tumor progression in cutaneous melanoma and rarely caused by exon 3 mutations. Mod. Pathol..

[58] Reifenberger J., Knobbe C.B., Wolter M., Blaschke B., Schulte K.W., Pietsch T., Ruzicka T., Reifenberger G. (2002). Molecular genetic analysis of malignant melanomas for aberrations of the WNT signaling pathway genes CTNNB1, APC, ICAT and BTRC. Int. J. Cancer.

[59] Jalaleddine N., El-Hajjar L., Dakik H., Shaito A., Saliba J., Safi R., Zibara K., El-Sabban M. (2019). Pannexin1 is associated with enhanced epithelial-to-mesenchymal transition in human patient breast cancer tissues and in breast cancer cell lines. Cancers (Basel).

[60] Schaefer K.N., Peifer M. (2019). Wnt/Beta-catenin signaling regulation and a role for biomolecular condensates. Dev. Cell.

[61] Krieghoff E., Behrens J., Mayr B. (2006). Nucleo-cytoplasmic distribution of beta-catenin is regulated by retention. J. Cell Sci..

[62] Grossmann A.H., Yoo J.H., Clancy J., Sorensen L.K., Sedgwick A., Tong Z.Z., Ostanin K., Rogers A., Grossmann K.F., Tripp S.R., Thomas K.R., D'Souza-Schorey C., Odelberg S.J., Li D.Y. (2013). The small GTPase ARF6 stimulates beta-catenin transcriptional activity during WNT5A-mediated melanoma invasion and metastasis. Sci. Signal..

[63] Serrat R., Lopez-Domenech G., Mirra S., Quevedo M., Garcia-Fernandez J., Ulloa F., Burgaya F., Soriano E. (2013). The non-canonical Wnt/PKC pathway regulates mitochondrial dynamics through degradation of the arm-like domain-containing protein Alex3. PLoS One.

[64] Godoy J.A., Arrazola M.S., Ordenes D., Silva-Alvarez C., Braidy N., Inestrosa N.C. (2014). Wnt-5a ligand modulates mitochondrial fission-fusion in rat hippocampal neurons. J. Biol. Chem..

[65] Cruz-Munoz W., Man S., Xu P., Kerbel R.S. (2008). Development of a preclinical model of spontaneous human melanoma central nervous system metastasis. Cancer Res..

[66] Shao Q., Lindstrom K., Shi R., Kelly J., Schroeder A., Juusola J., Levine K.L., Esseltine J.L., Penuela S., Jackson M.F., Laird D.W. (2016). A germline variant in the PANX1 gene has reduced channel function and is associated with multisystem dysfunction. J. Biol. Chem..

[67] Sacks D.B., Porter S.E., Ladenson J.H., Mcdonald J.M. (1991). Monoclonal-antibody to calmodulin - development, characterization, and comparison with polyclonal anticalmodulin antibodies. Anal. Biochem..

[68] Ran F.A., Hsu P.D., Wright J., Agarwala V., Scott D.A., Zhang F. (2013). Genome engineering using the CRISPR-Cas9 system. Nat. Protoc..

[69] White C.D., Li Z., Dillon D.A., Sacks D.B. (2011). IQGAP1 protein binds human epidermal growth factor receptor 2 (HER2) and modulates trastuzumab resistance. J. Biol. Chem..

