# Supplementary material for: Pannexin 1 binds β-catenin to modulate melanoma cell growth and metabolism
Source: J Biol Chem. 2021 Feb 26;296:100478. doi: 10.1016/j.jbc.2021.100478 (PMC8027267; doi:10.1016/j.jbc.2021.100478)
Supplement: Supplemental Data 1 [file mmc2.pdf]

COVID-19 is an emerging, rapidly evolving situation.
Get the latest public health information from CDC: https://www.coronavirus.gov .
Get the latest research from NIH: https://www.nih.gov/coronavirus.
Find NCBI SARS-CoV-2 literature, sequence, and clinical content: https://www.ncbi.nlm.nih.gov/sars-cov-2/.

BLAST ® » blastp suite » results for RID-FATPG3HV016

Job Title	Protein Sequence ...
RID	FATPG3HV016 Search expires on 06-27 11:09 am
Program	BLASTP
Database	nr
Query ID	lcl|Query_14447
Description	None ...
Molecule type	amino acid
Query Length	135

Descriptions

Description	Max Score	Total Score	Query Cover	E value	Per. Ident	Accession
pannexin-1 isoform X1 [Homo sapiens]	275	275	100%	5e-91	99.26%	XP_011541036.1
Pannexin 1 [Homo sapiens]	276	276	100%	1e-89	99.26%	AAH16931.1
pannexin-1 [Homo sapiens]	276	276	100%	1e-89	99.26%	NP_056183.2
Chain A, Pannexin-1 [Homo sapiens]	276	276	100%	1e-89	99.26%	6V6D_A
Chain A, Pannexin-1 [Homo sapiens]	276	276	100%	1e-89	99.26%	6LTN_A
Chain A, Pannexin-1 [Homo sapiens]	276	276	100%	1e-89	99.26%	6M66_A
pannexin-1 [Pan troglodytes]	273	273	100%	1e-88	97.79%	XP_522150.2
pannexin-1 [Gorilla gorilla gorilla]	273	273	100%	3e-88	97.79%	XP_004052028.2
Chain A, Pannexin-1 [Homo sapiens]	273	273	100%	3e-88	97.79%	6M67_A
pannexin-1 [Pan paniscus]	271	271	100%	6e-88	97.06%	XP_003813837.2
pannexin-1 [Hylobates moloch]	270	270	100%	3e-87	97.06%	XP_032023064.1
pannexin 1 [Homo sapiens]	268	268	100%	1e-86	97.79%	CAR31475.1
PREDICTED: pannexin-1 [Rhinopithecus bieti]	264	264	100%	4e-85	94.85%	XP_017729206.1
hypothetical protein EGK_06794 [Macaca mulatta]	264	264	100%	5e-85	94.85%	EHH23343.1
pannexin-1 isoform X1 [Theropithecus gelada]	264	264	100%	5e-85	94.85%	XP_025213190.1
PANX1 isoform 1 [Pongo abelii]	263	263	100%	2e-84	94.85%	PNJ86390.1
pannexin-1 [Macaca nemestrina]	262	262	100%	2e-84	94.12%	XP_011744164.1
pannexin-1 [Pongo abelii]	262	262	100%	2e-84	94.85%	NP_001124868.1
PREDICTED: pannexin-1 isoform X2 [Macaca fascicularis]	262	262	100%	3e-84	94.12%	XP_005579427.1
PREDICTED: pannexin-1 isoform X5 [Cercocebus atys]	261	261	100%	3e-84	93.38%	XP_011933209.1
pannexin-1 [Papio anubis]	262	262	100%	3e-84	94.12%	XP_003910603.2
pannexin-1 [Piliocolobus tephrosceles]	262	262	100%	3e-84	94.12%	XP_023081403.1
pannexin 1 [Homo sapiens]	262	262	100%	4e-84	96.32%	AAK91713.1
unknown [Homo sapiens]	262	262	100%	4e-84	96.32%	AAC61779.1
pannexin-1 [Nomascus leucogenys]	261	261	100%	6e-84	94.12%	XP_003253050.2

Description	Max Score	Total Score	Query Cover	E value	Per. Ident	Accession
PREDICTED: pannexin-1 isoform X1 [Mandrillus leucophaeus]	261	261	100%	8e-84	93.38%	XP_011850409.1
pannexin-1 [Macaca mulatta]	261	261	100%	9e-84	93.38%	XP_001088479.1
PREDICTED: pannexin-1 isoform X3 [Cercocebus atys]	261	261	100%	9e-84	93.38%	XP_011933207.1
PREDICTED: pannexin-1 isoform X1 [Macaca fascicularis]	262	262	100%	1e-83	94.12%	XP_015291098.1
pannexin-1 [Trachypithecus francoisi]	261	261	100%	1e-83	93.38%	XP_033063736.1
PREDICTED: pannexin-1 [Chlorocebus sabaeus]	260	260	100%	2e-83	93.38%	XP_008018745.1
pannexin 1 [Homo sapiens]	258	258	100%	2e-83	95.59%	AAK73361.1
PREDICTED: pannexin-1 isoform X2 [Cercocebus atys]	261	261	100%	3e-83	93.38%	XP_011933206.1
PREDICTED: pannexin-1 isoform X1 [Cercocebus atys]	261	261	100%	4e-83	93.38%	XP_011933205.1
pannexin-1 [Aotus nancymaae]	257	257	100%	3e-82	93.43%	XP_021521311.1
PREDICTED: LOW QUALITY PROTEIN: pannexin-1 [Callithrix jacchus]	253	253	100%	1e-80	91.97%	XP_003734197.1
pannexin-1 isoform X2 [Theropithecus gelada]	253	253	100%	1e-80	92.65%	XP_025213191.1
PREDICTED: pannexin-1 [Saimiri boliviensis boliviensis]	252	252	100%	4e-80	91.97%	XP_010350593.1
PREDICTED: pannexin-1 isoform X2 [Colobus angolensis palliatus]	251	251	100%	8e-80	91.91%	XP_011781953.1
PANX1 isoform 2 [Pongo abelii]	250	250	100%	2e-79	92.65%	PNJ86391.1
PREDICTED: pannexin-1 isoform X2 [Mandrillus leucophaeus]	249	249	100%	2e-79	91.18%	XP_011850410.1
PANX(HCVp) [Expression vector pcDNA-PANX(HCVp)]	247	247	100%	2e-78	91.18%	AQY63021.1
pannexin-1 [Sapajus apella]	245	245	100%	2e-77	90.51%	XP_032151649.1
PREDICTED: pannexin-1 [Cebus capucinus imitator]	244	244	100%	3e-77	90.51%	XP_017353902.1
pannexin-1 [Otolemur garnettii]	243	243	100%	1e-76	86.86%	XP_003780866.1
pannexin-1 [Microcebus murinus]	237	237	97%	2e-74	87.31%	XP_020142432.1
Pannexin-1 [Heterocephalus glaber]	235	235	100%	3e-74	84.67%	EHB16236.1
pannexin-1 isoform X2 [Heterocephalus glaber]	236	236	100%	4e-74	84.67%	XP_021108314.1
pannexin-1 isoform X1 [Heterocephalus glaber]	236	236	100%	7e-74	84.67%	XP_004838102.1
pannexin-1 [Ictidomys tridecemlineatus]	234	234	100%	2e-73	85.29%	XP_005337247.1
pannexin-1 [Peromyscus leucopus]	234	234	100%	2e-73	82.48%	XP_028715423.1
PREDICTED: pannexin-1 [Galeopterus variegatus]	234	234	99%	3e-73	85.29%	XP_008570699.1
PREDICTED: pannexin-1 [Propithecus coquereli]	234	234	97%	4e-73	85.82%	XP_012515514.1
PREDICTED: pannexin-1 [Chinchilla lanigera]	234	234	100%	5e-73	83.94%	XP_005379739.1
pannexin-1 isoform c [Rattus norvegicus]	231	231	100%	8e-73	81.02%	NP_001257477.1
pannexin-1 [Marmota flaviventris]	233	233	100%	8e-73	85.29%	XP_027807195.1
pannexin 1c [Rattus norvegicus]	231	231	100%	8e-73	81.02%	ADM92601.1
PREDICTED: pannexin-1 [Marmota marmota marmota]	233	233	100%	8e-73	85.29%	XP_015345468.1

Description	Max Score	Total Score	Query Cover	E value	Per. Ident	Accession
pannexin-1 isoform X1 [Urocitellus parryii]	233	233	100%	1e-72	84.56%	XP_026251872.1
PREDICTED: pannexin-1 [Orycteropus afer afer]	233	233	100%	1e-72	82.61%	XP_007936252.1
pannexin-1 [Tupaia chinensis]	233	233	100%	1e-72	83.94%	XP_014448799.1
PREDICTED: pannexin-1 [Chrysochloris asiatica]	232	232	100%	1e-72	83.33%	XP_006871259.1
PREDICTED: pannexin-1 [Manis javanica]	230	230	100%	2e-72	83.94%	XP_017501925.1
pannexin-1 [Carlito syrichta]	231	231	100%	2e-72	83.21%	XP_008058816.1
pannexin-1 [Rattus rattus]	232	232	100%	2e-72	81.75%	XP_032765552.1
PREDICTED: pannexin-1 [Jaculus jaculus]	231	231	100%	3e-72	81.75%	XP_004661987.1
Pannexin-1 [Camelus dromedarius]	228	228	99%	3e-72	85.29%	KAB1272795.1
PREDICTED: pannexin-1 [Peromyscus maniculatus bairdii]	231	231	100%	5e-72	81.02%	XP_006990587.1
PREDICTED: pannexin-1 [Dipodomys ordii]	230	230	100%	5e-72	83.21%	XP_012884270.1
pannexin-1 [Vicugna pacos]	231	231	99%	5e-72	86.03%	XP_006206811.1
pannexin-1 isoform X1 [Octodon degus]	231	231	100%	6e-72	83.21%	XP_004644824.1
pannexin-1 [Cavia porcellus]	230	230	100%	9e-72	81.75%	XP_003468590.1
pannexin-1 isoform a [Rattus norvegicus]	230	230	100%	9e-72	81.02%	NP_955429.1
pannexin-1 isoform X1 [Equus caballus]	230	230	100%	1e-71	83.94%	XP_001491337.3
pannexin-1 [Camelus dromedarius]	229	229	99%	2e-71	85.29%	XP_010987203.1
PREDICTED: pannexin-1 [Camelus bactrianus]	229	229	99%	3e-71	85.29%	XP_010971655.1
hypothetical protein A6R68_08941 [Neotoma lepida]	229	229	100%	4e-71	79.56%	OBS59938.1
pannexin-1 [Balaenoptera acutorostrata scammoni]	228	228	100%	5e-71	83.21%	XP_007189872.1
hypothetical protein E2I00_007542 [Balaenoptera physalus]	228	228	100%	6e-71	83.21%	KAB0389341.1
pannexin-1 [Mesocricetus auratus]	228	228	100%	6e-71	80.29%	XP_005084090.1
pannexin-1 [Pontoporia blainvillei]	223	223	100%	7e-71	83.21%	NIG58308.1
pannexin-1 [Grammomys surdaster]	228	228	100%	7e-71	80.29%	XP_028619221.1
pannexin-1 [Microtus ochrogaster]	228	228	100%	8e-71	79.56%	XP_005371751.1
pannexin-1 [Meriones unguiculatus]	228	228	100%	9e-71	80.29%	XP_021490828.1
pannexin-1 [Cricetulus griseus]	228	228	100%	1e-70	80.29%	XP_003506532.1
PREDICTED: pannexin-1 isoform X1 [Ceratotherium simum simum]	227	227	100%	1e-70	84.67%	XP_004427496.1
pannexin-1 [Lagenorhynchus obliquidens]	227	227	100%	1e-70	83.94%	XP_026943550.1
pannexin-1 [Orcinus orca]	227	227	100%	1e-70	83.94%	XP_004265516.1
pannexin-1 [Tursiops truncatus]	227	227	100%	2e-70	83.94%	XP_004327485.3
Pannexin-1 [Eschrichtius robustus]	225	225	100%	2e-70	82.48%	MBW00675.1
Hypothetical predicted protein [Marmota monax]	233	426	100%	2e-70	85.29%	VTJ85694.1
pannexin-1 [Loxodonta africana]	228	228	100%	3e-70	78.99%	XP_023410468.1
pannexin-1 [Neophocaena asiaeorientalis asiaeorientalis]	226	226	100%	5e-70	83.94%	XP_024587045.1

Description	Max Score	Total Score	Query Cover	E value	Per. Ident	Accession
PREDICTED: pannexin-1 [Elephantulus edwardii]	226	226	99%	5e-70	82.48%	XP_006902427.1
PREDICTED: LOW QUALITY PROTEIN: pannexin-1 [Equus przewalskii]	225	225	98%	8e-70	83.70%	XP_008537050.1
pannexin-1 [Mus caroli]	225	225	100%	9e-70	78.83%	XP_021028551.1
pannexin-1 [Pteropus vampyrus]	225	225	96%	9e-70	84.73%	XP_011373055.1
PREDICTED: LOW QUALITY PROTEIN: pannexin-1 [Equus asinus]	226	226	98%	1e-69	83.70%	XP_014690900.1
LOW QUALITY PROTEIN: pannexin-1-like [Castor canadensis]	223	223	98%	2e-69	82.96%	XP_020009467.1
Pannexin-1 [Pteropus alecto]	224	224	96%	2e-69	83.97%	ELK16487.1

Graphic Summary
